# A systematic review of epidemiology and outcomes of Crohn’s disease-related enterocutaneous fistulas

**DOI:** 10.1097/MD.0000000000030963

**Published:** 2022-11-11

**Authors:** Kristy Iglay, Dimitri Bennett, Michael D. Kappelman, Kamika Reynolds, Molly Aldridge, Chitra Karki, Suzanne F. Cook

**Affiliations:** a CERobs Consulting, LLC, Chapel Hill, NC; b Takeda Pharmaceuticals, Cambridge, MA; c Perelman School of Medicine, University of Pennsylvania, Philadelphia, PA; d Pediatric Gastroenterology, University of North Carolina, Chapel Hill School of Medicine, Chapel Hill, NC; e Department of Epidemiology, Gillings School of Global Public Health, University of North Carolina at Chapel Hill, Chapel Hill, NC.

## Abstract

Enterocutaneous fistulas (ECF) are rare sequelae of Crohn’s disease (CD) that occur either postoperatively or spontaneously. ECFs are associated with high morbidity and mortality. This systematic literature review assesses the disease burden of CD-related ECF and identifies knowledge gaps around incidence/prevalence, treatment patterns, clinical outcomes, healthcare resource utilization (HCRU), and patient-reported outcomes (PROs).

**Methods:**

English language articles published in PubMed and Embase in the past 10 years that provided data and insight into the disease burden of CD-related ECF (PROSPERO Registration number: CRD42020177732) were identified. Prespecified search and eligibility criteria guided the identification of studies by two reviewers who also assessed risk of bias.

**Results:**

In total, 582 records were identified; 316 full-text articles were assessed. Of those, eight studies met a priori eligibility criteria and underwent synthesis for this review. Limited epidemiologic data estimated a prevalence of 3265 persons with ECF in the USA in 2017. Clinical response to interventions varied, with closure of ECF achieved in 10% to 62.5% of patients and recurrence reported in 0% to 50% of patients. Very little information on HCRU is available, and no studies of PROs in this specific population were identified.

**Conclusion:**

The frequency, natural history, and outcomes of ECF are poorly described in the literature. The limited number of studies included in this review suggest a high treatment burden and risk of substantial complications. More robust, population-based research is needed to better understand the epidemiology, natural history, and overall disease burden of this rare and debilitating complication of CD.

## 1. Introduction

Crohn’s disease (CD) is a transmural inflammatory bowel disease with an undetermined etiology and an incidence and prevalence that is increasing substantially around the world, including an estimated 780,000 people in the USA.^[1–4]^ CD can have a debilitating impact on quality of life, particularly in the approximately 35% of patients who develop the complication of CD-related fistulas.^[3]^ Most observational and randomized studies of fistulas in patients with CD have focused on perianal fistulas, which account for roughly 20% to 65% of fistula occurrences.^[5]^ Enterocutaneous fistulas (ECFs) are rare, non-perianal fistulas connecting an abdominal portion of the gastrointestinal tract and the skin, and constitute approximately 6% of fistula cases in patients with CD.^[6]^ ECFs may occur owing to trauma or surgery, with complications often resulting from anastomotic leaks or an unrecognized bowel injury; however, they can also occur spontaneously.^[7]^

ECFs are associated with high morbidity and mortality,^[6,8]^ with mortality estimates ranging from 6% to 33%.^[3,8]^ Patients often experience septic complications, malnutrition, and substantial skin damage, as well as negative psychological effects.^[8,9]^ High initial fistula output, complications, comorbidities, low albumin level, and age are all factors that contribute to ECF outcomes.^[8]^

Treatment varies according to ECF type, but generally focuses on ***s***epsis control and ***s***kin care, ***n***utrition support, definition of intestinal ***a***natomy, and development of a surgical ***p***rocedure (often referred to as SNAP).^[8]^ Advances in the development of biologic drugs provide a potential alternative for surgery,^[10]^ and postoperative ECFs can respond to non-surgical interventions such as medication and skincare.^[5]^ Spontaneous ECFs, however, often require definitive surgical repairs as they rarely respond to medical therapy.^[5]^ There continues to be a substantial unmet need for novel therapeutic options for this condition.^[3]^

The objective of this systematic literature review was to summarize available evidence regarding the disease burden for patients with Crohn’s-related ECF, specifically centered around incidence and prevalence, treatment patterns, clinical outcomes, healthcare resource utilization (HCRU)/costs, and patient-reported outcomes (PROs). A detailed synthesis of the existing epidemiological literature is provided in this review and evidence gaps are highlighted.

## 2. Methods

### 2.1. Search strategy and selection criteria

The systematic literature review was performed as outlined in the Preferred Reporting Items for Systematic Reviews and Meta-Analyses (PRISMA) statement for systematic reviews and meta-analyses.^[11]^ The protocol for this review was registered with the International Prospective Register of Systematic Reviews (PROSPERO; registration number CRD42020177732). Ethical approval was not necessary as this study included only review of previously published studies.

Two electronic data sources (PubMed and Embase) were used to identify studies published within 10 years of the search date, March 25, 2020, using a search strategy that utilized terms for fistula type (ECF is included in this publication), CD, observational studies, treatments, patient outcomes, and HCRU/costs. All terms were searched using title/abstract and relevant Medical Subject Headings (MeSH) terms in PubMed and title/abstract/keyword and database-recommended candidate terms in Embase (Supplemental Digital Content [Table S1 http://links.lww.com/MD/H519 and Table S2 http://links.lww.com/MD/H520]). A manual search of key publications and references was also conducted.

The titles and abstracts of studies identified through the search were independently screened by two reviewers trained in epidemiology and conducting systematic literature reviews, in order to determine whether articles met the population, intervention, comparison, outcomes, time, and study design (PICOTS) criteria (Supplemental Digital Content [Table S3, http://links.lww.com/MD/H521]).^[12]^ Articles meeting these criteria were included in a full-text review phase, where they were independently assessed by each reviewer to determine eligibility for data abstraction. Any discrepancies between the reviewers at screening or full-text review steps were resolved by consensus. If consensus could not be achieved, a third trained reviewer was consulted to resolve any disagreements. Studies that were considered out of scope, or for which the full-text article was unavailable, and the abstract did not include the necessary information on methods and results, were excluded, with the rationale for exclusion documented. Data from each eligible study were independently abstracted by two reviewers using a standardized data abstraction form. Both reviewers jointly examined the abstraction spreadsheets in order to synthesize the abstracted data into one master spreadsheet. Data were extracted for a range of variables, including study type and design, population, outcomes, and limitations.

Studies included in the final abstraction for this report met the following criteria: reported on ECF separately from other types of fistula; an observational study (i.e., case-control, cohort/registry, or cross-sectional study); measured one of the outcomes of interest (incidence/prevalence, treatment patterns, clinical outcomes [e.g., healing/failure/ recurrence rates, postoperative infection], HCRU/costs, and PROs [e.g., Crohn’s Disease Activity Index (CDAI), Inflammatory Bowel Disease Questionnaire (IBDQ), 5-dimension EuroQol questionnaire (EQ-5D), pain]); and original research. Full eligibility requirements for study inclusion are shown in Table S3, http://links.lww.com/MD/H521. Case series were designated as cohort studies only if they met all of the following prespecified criteria: >10 patients; sampling based on patient exposure only (not outcome); outcome assessed over a prespecified follow-up period or mean/median follow-up reported; information available to calculate the absolute/relative risk; and sampling labeled as “consecutive” or text indicated all eligible patients were included to avoid selection of unique cases.

### 2.2. Risk of bias assessment

Two reviewers independently evaluated risk of bias in each article using the Risk of Bias in Non-randomized Studies of Interventions (ROBINS-I) tool for observational studies.^[13]^ Disagreements were resolved with consensus or a consultation with a third reviewer when consensus could not be achieved.

## 3. Results

### 3.1. Systemic literature review

**Figure 1.**
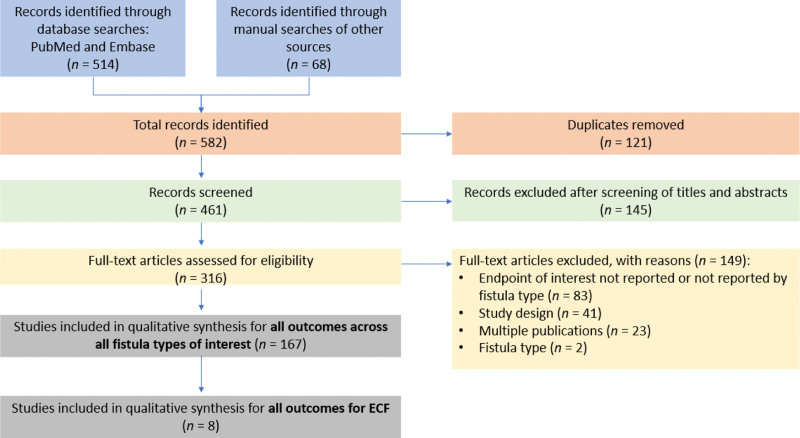
PRISMA flow diagram. ECF = enterocutaneous fistulas, PRISMA = Preferred Reporting Items for Systematic Reviews and Meta-Analyses.

The electronic search returned 514 articles; an additional 68 were identified from a hand search of other sources; 121 duplicates were deleted. Of the 461 records screened based on their titles and abstracts, 316 were included in the full-text assessment. Of those, 149 were excluded based on the inclusion/exclusion criteria (Fig. 1). In total, eight studies were included for final qualitative synthesis for CD-related ECF. One paper addressed spontaneous ECF, three papers both spontaneous and postoperative fistula, and four papers did not specify ECF subtype (Table 1).

**Table 1:** Characteristics of papers included in systematic literature review (n = 8 studies).

Author, Year	Country	Inclusion criteria	Exclusion criteria	Postoperative or spontaneous ECF	Intervention(s)	Risk of bias (ROBINS-I)
Amiot 2014^[10]^	France, Belgium	Anti-TNF-treated CD patients with ECF treated by gastroenterologists who were part of GETAID (12 sites in France and Belgium) between January 2000 and December 2009:	Perianal fistula	Both	All patients treated with anti-TNF treatment	Moderate
		• ECF developed on a segment of bowel affected by CD			Infliximab, 79% (38/48)	
		• Patients with concomitant abdominal abscess were included only if sepsis resolved under antibiotics associated with the drainage of the infected collection			Adalimumab, 8% (4/48)	
		• All patients had active disease			Infliximab followed by adalimumab, 13% (6/48)	
Boyle 2015^[14]^ (abstract only)	Ireland	CD patients with history of ECF or treated for ECF over a 36-yr period who were part of a database affiliated with St. Vincent’s University Hospital in Dublin	Not reported	Both: 22% postop, 78% spontaneous	14% (6*/41) treated medically alone86% (35*/41) proceeded to surgery	Moderate
Gyorki 2010^[17]^	Australia	Patients who had ECF at an unidentified single tertiary center	Unless there was an associated enterocutaneous component:	Not reported	Resection and re-anastomosis: 100% (n = 10)	Moderate
			• Enteroenteric fistula;			
			• Enterovaginal fistula;			
			• Enterovesical fistula;			
			• Peristomal fistula;			
			• Perianal fistula			
Li 2014^[19]^	China	• CD patients with non-perianal fistula who underwent elective bowel resections for ECFs at the study hospital (Jinling Hospital, Nanjing) between February 2001 and April 2011	• Had undergone temporal enterostomy rather than definitive operation for resection of fistulas	Not reported	3-mo EEN group with exclusion of a normal diet 3 mo pre-operatively: n = 55 (45%,* 55/123)	Moderate
		• The diagnosis of CD was made on the basis of the clinical, endoscopic, histological and radiological criteria according to Lennard-Jones	• Had undergone emergency surgeries and operations for perianal disease		Non-EEN group: n = 68 (55%,* 68/123)	
Ravindran 2014^[6]^	Australia	Patients who underwent definitive surgery for cure of an ECF (defined as a fistulous communication to the skin originating from the small bowel, colon, or an anastomosis) within the colorectal unit of Royal Prince Alfred Hospital, Sydney, between January 1, 2000 and December 31, 2010	• Patients with an internal or perianal fistula • Patients whose ECF closed with conservative management	Spontaneous	Patients undergoing:Resection (n = 42 procedures)†Wedge resection (n = 2 procedures) †	Moderate
Schwartz 2019^[15]^	USA	Cases of CD (at least one claim of CD-related ICD-9 code 555.0, 555.1, 555.2, and 555.9 in recent 5-yr history) identified through December 31, 2014 with codes for fistulizing disease (identified by ICD-9 and surgical codes); Truven Health MarketScan Database	Not reported	Not reported	Not applicable	Moderate
Yan 2014^[2]^	China	Patients with CD and a diagnosis of ECF in Department of Surgery, Jinling Hospital, Medical School of Nanjing University, Nanjing who were treated with short peptide-based EN for 3 mo between May 2007 and March 2012	• Withdrawal from EN therapy; • Upcoming operations in the next 3 mo; • Biological therapies or corticosteroids in previous 8 wk; • Intestinal stenosis; • Serious infection;	Both	Short peptide-based EN (30 Kcal/kg per d) by continuous infusion through a nasogastric feeding tube for 3 mo: 100% (n = 48)	Moderate
			• Intestinal-visceral fistula; • Intestinal-intestinal fistula; • Patients who were assigned to an operation during the study period because of significantly reduced intra-abdominal adhesion;			
			• Internal fistula; • Intestinal stenosis; • Patients showed high output (>200 mL/24 h); • Death; • Perianal fistulas			
Yoon 2010^[18]^	South Korea	Consecutive patients who underwent laparotomy and were proven by pathologists with CD in the Department of Colon & Rectal Surgery, Asan Medical Center between October 1991 and December 2008	Patients with only anorectovaginal fistulas	Not reported	Cases – Crohn’s patients with ≥ 1 intra-abdominal fistula: 32.7% (83/254) patientsControls – Crohn’s patients without intra-abdominal fistula: 67.3%* (171/254) patientsNumber of surgeries (n = 270)Cases: 93	Moderate
					Controls (i.e., cases without fistula): 177Among the 93 surgical procedures performed in the cases: • Ileocecal resection, 29%‡ • Right hemicolectomy, 26.9%‡ • Small bowel resection and anastomosis, 22.6%‡	
					• Total colectomy, 13%‡ • Intestinal resection, 95.7%‡ oPrimary anastomosis, 82.8% (77/93) oTemporary or permanent stoma, 16.1%* (15/93)	

CD = Crohn’s disease, ECF = enterocutaneous fistulas, EN = enteral nutrition, GETAID = Groupe d’Etude Thérapeutique des Affections Inflammatoires Digestives, ICD-9 = International Classification of Diseases = Ninth Revision, ROBINS-I = Risk of Bias in Non-randomized Studies of Interventions, TNF = tumor necrosis factor.

* Calculated value.

† Includes patients with ECF ± CD (n = 16 for CD ECF, procedures not reported by etiology).

‡ Numbers for calculation not reported.

Seven of the eight studies were retrospective cohort studies or case series that met the review definition for cohort studies. One study was a prospective cohort study.^[2]^ Two of the eight studies were databases analyses,^[14,15]^ and the remainder came from either single- or multi-site, surgical center-based studies. All eight papers were identified as having a moderate risk of bias (Table 1).

### 3.2. Incidence and prevalence

One paper was identified that provided a population-based estimate of ECF incidence or prevalence during the 10-year search period (Table 2). The authors conducted a claims-based study using the Truven Health MarketScan database to identify cases of CD with codes for fistulizing disease and identified that 3265 patients were affected by ECF in the USA in 2017.^[15]^ As part of the study, the authors also conducted a systematic literature review. Using data from two articles,^[3,16]^ the authors estimated the prevalence of patients with 1, 2, 3, 4, and 5 episodes of CD-related ECF as 65.4%, 19.2%, 8.2%, 4.5%, and 2.7%, respectively. The median duration of the fistula episode for patients with 1, 2, 3, 4, and 5 episodes was 0.5, 3.9, 7.2, 10.5, and 13.8 years, respectively. The weighted average of median duration of fistulizing CD ECF was 2.5 years.^[15]^ The study has several notable limitations, including possible misclassification and underreporting of diagnosis and procedures in claims, as well as lack of validation of the International Classification of Diseases (ICD) and procedure codes used. No specific ICD-9 or surgical codes were available for the ECF calculations; the number of records coded for “intestinal fistulas excluding the rectum/anus” was multiplied by 19%, the proportion of these fistulae that were classified as ECF in a prior study from 2002,^[3]^ which could lead to an overestimation of cases if more recent improvements in care have reduced fistula occurrence. A further limitation is the lack of reporting of spontaneous vs. postoperative ECFs.

**Table 2:** Prevalence of Crohn’s-related enterocutaneous fistulas (n = 1 study).

Author, Year	Study/base population	Incidence	Prevalence
Schwartz 2019^[15]^	• Cases of CD with codes for fistulizing disease (Truven Health MarketScan) • Age not reported • n = 73,878 (95% CI: 72,203–75,553) for 2014 • n = 75,666 (95% CI: 73,950–77,382) for 2017 • USA (data up to 2014)	Not reported	From Schwartz database analysis:2014 prevalence = 3188 (95% CI: 2960–3416)2017 projected prevalence = 3265 (95% CI: 3031–3499)

CD = Crohn’s disease, CI = confidence interval.

Two studies provided insight into the distribution of spontaneous versus postoperative ECFs in patients with CD.^[10,14]^ In these studies, the percentage of patients with CD whose ECFs occurred postoperatively were 29.2% (multicenter retrospective cohort study)^[10]^ and 22% (retrospective cohort study based on a prospectively maintained inflammatory bowel disease database).^[14]^

### 3.3. Treatment patterns

The current review included seven (non-population-based) studies that addressed treatment patterns among patients with ECF (Tables 3 and 4). Treatments for ECF evaluated in these papers included medical management (biologics and CD medications), use of enteral and parenteral nutrition, and surgical procedures (e.g., resection, re-anastomosis, and end ileostomy). It should be noted that some of the studies identified describe older cohorts that are perhaps not representative of current treatment options, particularly with respect to use of biologics.^[14,17,18]^

**Table 3:** Studies providing information on treatment patterns (not including enteral nutrition) (n = 5 studies).

Author, Year	Baseline operations	Distribution of surgeries of interest	Surgery for failures/recurrence during follow-up	Immunosuppressive agents	Antibacterial agents
Amiot 2014^[10]^	Had previous surgery:Overall, 92% (44/48)Spontaneous ECF, 88% (30/34)Postop ECF, 100% (14/14)	Not applicable	Surgery after the occurrence of post–anti-TNF abscess: 27%* (13/48), with a median delay of 4.6 (2.6–24.9) moTreated within 3 mo: 4Treated after 3 mo: 96.3%* (3/48) needed surgery after relapse	Prior medications:Treatment history, Overall:Immunosuppressants, 63% (30/48)Anti-TNF, 33% (16/48)Treatment history, spontaneous ECF:Immunosuppressants, overall, 65% (22/34)	Pre-intervention:Not reportedPost-intervention:31% (15/48) were treated medically with percutaneous drainage and antibiotics for the occurrence of post–anti-TNF abscess (time duration not reported). Two achieved a complete ECF closure within 3 mo.
			At the end of follow-up, 54% (26/48) needed surgery after a median delay of 15.6 (7.2–36.2) mo58% (15/26) had ECF31% (8/26) had underlying intestinal stricture11% (3/26) had abdominal abscess	Anti-TNF, 27% (9/34)Treatment history, postop ECF:Immunosuppressants, overall, 57% (8/14)Anti-TNF, 50% (7/14)Current medications:Anti-TNF, overall population:	
			27% (7/26) required a temporary stoma after resection to prevent early ECF recurrence	Infliximab, 79% (38/48)Adalimumab, 8% (4/48)Infliximab followed by adalimumab, 13% (6/48)Anti-TNF, spontaneous ECF:Infliximab, 82% (28/34)Adalimumab, 6% (2/34)	
				Infliximab followed by adalimumab, 12% (4/34)Anti-TNF, postoperative ECF:Infliximab, 72% (10/14)Adalimumab, 14% (2/14)Infliximab followed by adalimumab, 14% (2/14)Note: Anti-TNF therapy duration not able to be reported because authors do not separate ≥6 mo and >6 mo in Table 3	
				Concomitant immunomodulator therapy:Overall population, 77% (37/48)Spontaneous ECF, 79% (27/34)Postop ECF, 71% (10/14)Concomitant steroids:	
				Overall population, 15% (7/48)Spontaneous ECF, 9% (3/34)Postop ECF, 29% (4/14)Postoperative Crohn’s medications:Not reported	
Boyle 2015^[14]^	Not reported	86% (35*/41) of patients were initially managed medically and proceeded to surgery14% (6*/41) were medically managed only	Not reported	Overall:Biologics, 29% (12/41)Preoperative:Biologics, 14.6%* (6/41)Postoperative:	Preoperative:Not reportedPostoperative:Not reported
				Biologics, not reported for this specific timeframe, but can assume an additional six began treatment postoperatively to sum to a total of 12 patients treated with biologics during the study: 14.6%* (6/41)Mean time from surgery to postoperative biologic treatment: 3.5 yr	
Gyorki 2010^[17]^	Not reported by fistula type	Resection and re-anastomosis: 100% (n = 10)Two Crohn’s patients had an end ileostomy fashioned	Not reported by fistula type	Preoperative:Not reportedPostoperative:Not reported	Preoperative:Not reportedPostoperative:Not reported by fistula type
Ravindran 2014^[6]^	Not reported by fistula type	Not reported by fistula type	Two recurrences occurred in patients with Crohn’s disease at 3 mo. Among them: • one patient required further surgical closure • one patient closed on conservative management	Pre-intervention:Not reported by fistula typePost-intervention:Not reported	Pre-intervention:Not reportedPost-intervention:Not reported
Yoon 2010^[18]^	Not reported by fistula type	Not reported by fistula type	None	Pre-intervention:Not reportedPost-intervention:Among the 6 ECF that recurred clinically: • Infliximab, 66.7%* (4/6) • Managed conservatively, 33.3%* (2/6)	Pre-intervention:Not reported by fistula typePost-intervention:Not reported

ECF = enterocutaneous fistulas, TNF = tumor necrosis factor.

* Calculated value.

**Table 4:** Studies providing information on treatment patterns: enteral nutrition (n = 2 studies).

Author, Year	Baseline operations	Distribution of surgeries of interest	Surgery for failures/recurrence during follow-up	Immunosuppressive agents				Antibacterial agents
Li 2014^[19^	The sump drained, a modified closed double-lumen irrigation-suction tube was applied to both groups in the preoperative 3-mo period for surgical preparation	Patients received EEN with exclusion of a normal diet for 3 mo pre-operatively: n = 55 (45%,* 55/123)Non-EEN group: n = 68 (55%,* 68/123)	Not reported	Both groups were administered oral *Tripterygium wilfordii* Hook F (TWP), a Chinese herbal medicine with reported immunosuppressive activity • TWP pre-operatively:				Preoperative:Not reportedPostoperative:Not reported
				Group	n	Yes n (%)	No n (%)	
				All patients	123	61 (49.6%)	62 (50.4%)	
				EEN group	55	22 (40.0%)	33 (60.0%)	
				Non-EEN group	68	39 (57.4%)	29 (42.6%)	
				• TWP postoperatively:				
				Group	n	Yes n (%)	No n (%)	
				All patients	123	75 (61.0%)	48 (39.0%)	
				EEN group	55	35 (63.6%)	20 (36.4%)	
				Non-EEN group	68	40 (58.8%)	28 (41.2%)	
				• Preoperative corticosteroids:				
				Group	n	Yes n (%)	No n (%)	
				All patients	123	8 (6.5%)	115 (93.5%)	
				EEN group	55	2 (3.6%)	53 (96.4%)	
				Non-EEN group	68	6 (8.8%)	62 (91.2%)	
				• Postoperative Crohn’s medications: Both groups were administered oral mesalazine after discharge				
Yan 2014^[2]^	Previous appendectomy: • Colonic fistula patients, 71.4%* (10/14) • Small intestine fistula patients, not reported • Ileocolic anastomosis fistula patients, not reported	Short peptide-based EN (30 Kcal/kg per d) by continuous infusion through a nasogastric feeding tube for 3 mo: 100% (n = 48)	Among the eight patients who relapsed postoperatively: • Bowel resection surgery, 100%* (8/8)	Baseline Crohn’s medications:5-aminosalicylic acidTotal, 66.7%* (32/48)Closed group, 66.7%* (20/30)Unclosed group, 66.7%* (12/18)ImmunosuppressantTotal, 33.3%* (16/48)Closed group, 33.3%* (10/30)Unclosed group, 33.3%* (6/18)Patients who received preoperative 5-aminosalicylic acid or immunosuppressive therapy remained on these medications during the 3-mo EN treatment periodPostoperative Crohn’s medications:Not reported				Baseline antibiotic use:Total, 52.1%* (25/48)Closed group, 40.0%* (12/30)Unclosed group, 72.2%* (13/18)Post-intervention antibiotic use:Not reported

EEN = exclusive enteral nutrition, EN = enteral nutrition, TWP = *Tripterygium wilfordii* Hook F.

* Calculated value.

No natural history studies that prospectively collected data in a standardized manner at first presentation were identified; however, a notable feature of treatment journeys of patients with CD-related ECF is the documented need for multiple procedures and other therapies. For example, in a retrospective records review of patients with ECF followed by a French gastroenterology group, 54% (26/48) of patients needed surgery after a median of 15.6 (7.2–36.2) months following treatment with an anti-tumor necrosis factor (TNF) therapy. Fifteen of those patients (58%) required surgery for an ECF, 8 (31%) for an underlying intestinal stricture, and three (11%) for an abdominal abscess. Seven of those patients required a temporary stoma after resection to prevent early ECF recurrence.^[10]^

Two studies from China described use of exclusive enteral nutrition (EEN) in patients with ECF.^[2,19]^ Li et al^[19]^ evaluated the effects of preoperative 3-month EEN on the incidence of septic complications, while Yan et al^[2]^ evaluated ECF-related outcomes following 3 months of EEN. Li et al^[19]^ reported that, 49.6% of patients received an immunosuppressive herbal medicine and 6.5% received preoperative corticosteroids. Following a sump drain procedure, 61% received the herbal medicine and all patients (100%) received postoperative CD medications. In Yan et al,^[2]^ eight of 14 operative patients relapsed, and all received bowel resection surgery.

### 3.4. Clinical outcomes

Seven studies included data on clinical outcomes for treatments of ECF (Table 5). Most studies based outcome measures on success of interventions; however, it should be noted that authors used different terminologies to describe success and did not always provide clear definitions of their meanings. Five studies measured fistula closure/healing.^[2,6,10,14,17]^ Two studies measured maintenance of closure/recurrence,^[6,10]^ and three studies evaluated the need for follow-up surgery.^[10,14,18]^

**Table 5:** Studies providing information on clinical outcomes for treatments of ECF (n = 7 studies).

Author, Year	Crohn’s ECF sample size	Median follow-up duration (range)	Key outcomes measured and definitions	Success and failure rates	Postoperative infection rates
Amiot 2014^[10]^	48	3.0 yr (IQR 2.0–6.6 yr)	Complete ECF closure = complete closure of all fistulae at physical examination and complete cessation of the drainage from the fistula without occurrence of any abscess or need for surgery within the first 3 mo following anti-TNF therapyClinical response = reduction in the number of draining fistulae by at least 50% from baselineMaintenance of ECF closure at the end of follow-upOccurrence of abscessesNeed for surgery	Clinical response: 54% (26/48)Complete ECF closure at month 3: 33% (16/48)Age > 23 at CD diagnosis associated with complete ECF closure (*P* = .07, univariate):Age > 23 at CD diagnosis, 50.0%† (11/22)Age < 23 at CD diagnosis, 23.1%† (6/26)ECF closure at the end of follow-up: 17% (8/48)Need for surgery by the end of follow-up: 54% (26/48)	After anti-TNF therapy, an abdominal abscess developed in 31% (15/48) patientsAt the end of follow-up, 7%† (3/48) patients developed abdominal abscess3.8%* (1/26) of the 26 patients who needed surgery after anti-TNF therapy developed sepsis. The patient died after surgery from abdominal sepsis
				Probabilities of surgery (numbers for calculations not reported):1 yr, 41%2 yr, 46%3 yr, 59%50%† (8/16) patients who achieved complete ECF closure after 3 mo relapsed	
Boyle 2015^[14]^	41	Mean/median follow-up not reported, but follow-up was 6 mo for fistula healing rate	Outcomes included fistula control or healing, and among medically treated, no escalation to surgery.Definitions not reported	Overall fistula healing rate at 6 mo: 73% (30†/41)Among those treated with biologics pre-operatively or postoperatively, 25% (3/12) achieved fistula controlRecurrence rate: 0% among patients treated surgically	Not reported
Gyorki 2010^[17]^	10	Mean 37.3 mo(0.5–217 mo)	Spontaneous fistula closure not defined	Spontaneous fistula closure in Crohn’s ECF patients: 10% (1†/10)	Not reported by fistula type
Li 2014^[19]^	123	Mean/median follow-up not reported, but the routine duration of follow-up was a minimum of 3 mo, and the final follow-up ended 2 yr after operation, unless intra-abdominal septic complications occurred, or patients were lost	Rate of IASCs 3 mo after surgery and overall cumulative rate at the end of follow-upIASCs included anastomotic leakage, intra-abdominal abscesses and ECFs but they only reported anastomotic leakage and intra-abdominal abscesses after bowel resections for ECFs	Not reported	• Proportion of patients having intra-abdominal abscesses after bowel resections for ECFs (as part of intra-abdominal septic complications, IASCs): oIn all patients, 4.1% (5/123) oIn EEN group, 1.8% (1/55) oIn non-EEN group, 5.9% (4/68)
					*P* value from χ^2^ test for group comparison: 0.499 • Proportion of patients having ISACs (including anastomotic leakage and intra-abdominal abscesses) 3 mo after bowel resection for ECFs: oIn all patients, 11.4% (14/123) oIn EEN group, 3.6% (2/55) oIn non-EEN group, 17.6% (12/68)
					*P* value from χ^2^ test for group comparison: 0.020 • Proportion of patients having anastomotic leakage 3 mo after bowel resection for ECFs: oIn all patients, 7.3% (9/123) oIn EEN group, 1.8% (1/55) oIn non-EEN group, 11.8% (8/68) *P* value from χ^2^ test for group comparison: 0.079
Ravindran 2014^[6]^	16	Mean/median follow-up not reported, but all patients were followed up at 6–8 wk after discharge from hospital and thereafter as indicated	Successful closure = All ECFs remaining closed for at least 3 moRecurrence = reappearance of the ECF within those 3 mo	Successful closure not reported by fistula typeRecurrences at 3 mo: 12.5% (2/16) in patients with CDOf the two recurrences that occurred in patients with CD at 3 mo: • one patient required further surgical closure • one patient closed on conservative management	Intra-abdominal abscess at definitive closure:Overall, 62.5%† (10/16)Clavien ≥ III, 40.0%† (2/5)Clavien < III, 72.7%† (8/11)
Yan 2014^[2]^	48	Mean/median follow-up not reported, but all patients were followed up for at least 6 mo after EN therapy	Closed fistula = a covering by skin for at least 2 wk	• After 3 mo EN treatment, 62.5% (30/48) obtained a successful closure of fistula within an average time of 32.4 ± 8.85 d, including: o60.0%† (24/40) postoperative ECF within 34.1 ± 8.7 d o75.0%† (6/8) spontaneous ECF within 25.5 ± 5.6 d • Closure rate, by patient characteristics at baseline: SexMale, 67.6%† (23/34)Female, 50.0%† (7/14)Location of disease:Ileal, 68.8%† (11/16)	Not reported, but “there were no serious adverse events reported in all eligible patients”
				Colonic, 57.1%† (4/7)Ileocolonic, 60.0%† (15/25)Location of fistula:Small bowel fistula, 69.6%† (16/23)Colonic, 64.3%† (9/14)Ileocolic anastomosis, 45.5%† (5/11); 1 of them closed fistula by 3 moDrugs:	
				5-aminosalicylic acid, 62.5%† (20/32)Immunosuppressant, 62.5%† (10/16)Antibiotic, 48.0%† (12/25)Relapse rate:26.7%† (8/30) among the 30 patients who obtained closure of fistula. Times to relapse for the eight patients were at 3, 6, 10, 11, 13, 26, 33, and 44 mo after closure	
Yoon 2010^[18]^	28	Mean (± SE) 32.5 ± 3.1 mo (1–131 mo)	Surgical recurrence = cases that required a second laparotomy for recurrence of CD during the follow-up period	In the 28 CD patients with ECFs, 21.4%† (6/28) recurred clinically (no further laparotomies in these patients)Among the 6 ECFs that recurred clinically, 66.7%† (4/6) were treated successfully by infliximab	Not reported by fistula type

CD = Crohn’s disease, ECF = enterocutaneous fistulas, EEN = exclusive enteral nutrition, EN = enteral nutrition, IASC = intra-abdominal septic complication, IQR = interquartile range, SE = standard error, TNF = tumor necrosis factor.

† Calculated value.

“Closure” or “complete closure” of the ECF was reported in three studies,^[2,10,17]^ with rates ranging from 10% following resection and re-anastomosis^[17]^ to 62.5% of patients following enteral nutrition treatment.^[2]^ “Overall fistula healing rate” at 6 months was 73% in the one study that reported that outcome.^[14]^ Within that same cohort study, “fistula control” was achieved in 25% of patients treated with biologics pre- or postoperatively.^[14]^ An additional study showed that 54% of patients achieved “clinical response”, defined as reduction in number of draining fistulae following anti-TNF treatment.^[10]^

“Recurrence” or “relapse” was reported in five studies^[2,6,10,14,18]^ and rates ranged from 0% among patients treated with medical management or medical management followed by surgery^[14]^ to 50% in patients receiving anti-TNF treatment.^[10]^ Among the three studies reporting occurrence of abdominal abscess,^[6,10,19]^ the proportion of patients experiencing this complication ranged from 4.1% in patients undergoing bowel resection for ECF^[19]^ to 62.5% in patients undergoing resection or wedge resection.^[6]^

The wide range of results in these outcomes may be due to differences in actual efficacy of the various interventions or differences in study design, population, outcome definition, or other study-level factors. The variation in outcomes measured and ways in which they are defined prevents broad conclusions about the overall success and failure rates of interventions. However, the results reported in the limited number of studies available further demonstrate the high and sometimes multifaceted treatment burden among patients with ECF, overall.

### 3.5. Healthcare resource utilization

One of the eight studies reported HCRU, but did not report results separately for patients with CD- and non-CD-related ECF.^[6]^ Among patients with either CD- or non-CD-related ECF, median postoperative length of stay was 14 days (range, 2–213 days) and 32% of patients required postoperative intensive care unit admissions. More studies are needed to determine the direct and indirect costs of CD-related ECFs, including healthcare visits, hospitalizations, copays, prescriptions, and work and school productivity.

### 3.6. Patient-reported outcomes

This review did not identify studies that included data from prespecified PROs. More studies are needed to determine overall health-related quality of life, as well as the experience of pain and emotional health that could be captured uniquely through PROs.

## 4. Discussion

The current systematic literature review assessed the disease burden of patients with CD-related ECF, specifically centering around incidence/prevalence, treatment patterns, clinical outcomes, HCRU/costs, and PROs. Eight studies met the a priori criteria for inclusion and were synthesized to characterize and quantify the epidemiologic burden of non-perianal CD-related ECF. The burden of ECF for patients is substantial and can include skin irritation and excoriation, pain, malnutrition, and life-threatening infection.^[20]^ Intestinal failure is one of the most dangerous complications of ECF and can lead to significant morbidity and mortality.^[9]^

This review revealed a paucity of real-world evidence despite the high risk of morbidity and mortality due to sepsis and other complications. The review also identified several gaps in our knowledge of this disease and areas for further research that could provide clarity around the overall burden of ECF.

The incidence of ECF and associated complications (e.g., sepsis) is not well quantified. One administrative database study projected the 2017 prevalence of ECF to be 3265 persons in the USA,^[15]^ but no other studies providing robust estimates were identified. This knowledge gap should be addressed to better understand the number of patients at risk for substantial morbidity and mortality. Updated incidence and prevalence figures are particularly important when new treatments are being developed to evaluate the feasibility of clinical trials.

Additional knowledge gaps include the lack of information on risk factors and prognostic factors for ECF, as well as updated data on natural history and clinical and comparative effectiveness research. Treatments for ECF identified in this review included medical management, use of enteral nutrition, and surgical procedures. We observed high variability in response rates, with closure of the ECF achieved in 10% to 62.5% of patients, while recurrence or relapse occurred in a wide range (0–50%) of patients. The variability is likely due to differences in study population, design, interventions, and inclusion of heterogeneous outcomes; thus, broad statements about overall effectiveness of ECF treatments cannot be made at this time. Individual study limitations and the need for more complete data on the clinical events during follow-up warrant additional studies, which could be facilitated in part by electronic medical records.

Studies are also needed to determine the direct and indirect costs of ECF, particularly those relating to healthcare visits, copays, hospitalizations, prescriptions, ancillary care such as psychological support, missed days at work and/or school, and productivity loss.

This review has several strengths and limitations. Strengths include its compliance with established guidelines for systematic literature reviews, including the use of a prespecified protocol and search criteria. The protocol was registered with PROSPERO to promote transparency and allow for future replication or updates. Although this review was designed to capture a wide range of literature, it is limited to recent publications in the past 10 years, as well as publications in the English language, and may therefore be limited by a lack of representativeness for the full body of published literature. Generalizability is also limited to populations for which data have been reported. Most of the studies were clinic based and/or had small sample sizes. Also, three of the eight studies evaluated were limited by short follow-up. There was lack of confounding adjustment and outcomes were generally not well defined. Finally, there was a notable lack of studies reporting PROs, which would have provided valuable insight into the patient experience. Despite limitations, this review illuminates an overall picture of the burden of ECF and highlights several data gaps that can be addressed with further research.

## 5. Conclusion

This systematic review identified a small body of literature addressing the burden of CD-related ECFs, a rare and debilitating complication of CD. Substantial gaps in knowledge surrounding CDs-related ECF were described. Additional high-quality, real-world evidence is needed to better understand the epidemiology, treatment patterns, and clinical outcomes associated with ECF as well as HCRU/costs and PROs among this patient population.

## Acknowledgments

Sydney Thai, Sapna Rao, Courtney Schlusser, and Xinruo Zhang conducted the abstraction and quality assessment and provided valuable consultation for the writing of this manuscript. Oxford PharmaGenesis provided editorial assistance in the preparation of the manuscript.

## Author contributions

KI, MA, KR, and SFC were involved in the literature review design, performing the literature review, drafting of the report and manuscript, critical revision, and approval of the manuscript. MK was involved in the literature review design, critical revision, and approval of the manuscript. DB and CK were involved in the study concept, literature review design, drafting of the report and manuscript, critical revision, and approval of the manuscript.

**Conceptualization:** Kristy Iglay, Dimitri Bennett, Chitra Karki, Suzanne F. Cook.

**Data curation:** Kristy Iglay, Kamika Reynolds.

**Formal analysis:** Kristy Iglay, Kamika Reynolds.

**Investigation:** Kristy Iglay, Dimitri Bennett, Michael D. Kappelman, Chitra Karki.

**Methodology:** Kristy Iglay, Dimitri Bennett, Suzanne F. Cook.

**Project administration:** Kristy Iglay, Chitra Karki.

**Visualization:** Kristy Iglay.

**Writing – original draft:** Kristy Iglay, Dimitri Bennett, Michael D. Kappelman, Kamika Reynolds, Molly Aldridge, Chitra Karki, Suzanne F. Cook.

**Writing – review & editing:** Kristy Iglay, Dimitri Bennett, Michael D. Kappelman, Kamika Reynolds, Molly Aldridge, Chitra Karki, Suzanne F. Cook.

## Supplementary Material



## References

[1] Shivashankar R Tremaine W Harmsen S. Updated incidence and prevalence of Crohn’s disease and ulcerative colitis in Olmsted County, Minnesota (1970–2010): ACG IBD Research Award: 1687. Am J Gastroenterol. 2014;109:S499. 10.1016/j.cgh.2016.10.039 PMC5429988 27856364

[2] Yan D Ren J Wang G. Predictors of response to enteral nutrition in abdominal enterocutaneous fistula patients with Crohn’s disease. Eur J Clin Nutr. 2014;68:959–63. 24619104 10.1038/ejcn.2014.31

[3] Schwartz DA Loftus EV Jr. Tremaine WJ. The natural history of fistulizing Crohn’s disease in Olmsted County, Minnesota. Gastroenterology. 2002;122:875–80. 11910338 10.1053/gast.2002.32362

[4] Ng SC Shi HY Hamidi N. Worldwide incidence and prevalence of inflammatory bowel disease in the 21st century: a systematic review of population-based studies. Lancet. 2018;390:2769–78. 10.1016/S0140-6736(17)32448-0 29050646

[5] Gomez-Senent S Barreiro-de-Acosta M Garcia-Sanchez V. Enterocutaneous fistulas and Crohn’s disease: clinical characteristics and response to treatment. Rev Esp Enferm Dig. 2013;105:3–6. 23548004 10.4321/s1130-01082013000100002

[6] Ravindran P Ansari N Young CJ. Definitive surgical closure of enterocutaneous fistula: outcome and factors predictive of increased postoperative morbidity. Colorectal Dis. 2014;16:209–18. 24521276 10.1111/codi.12473

[7] Gribovskaja-Rupp I Melton GB. Enterocutaneous fistula: proven strategies and updates. Clin Colon Rectal Surg. 2016;29:130–7. 27247538 10.1055/s-0036-1580732 PMC4882173

[8] Gecse K Khanna R Stoker J. Fistulizing Crohn’s disease: diagnosis and management. United European Gastroenterol J. 2013;1:206–13. 10.1177/2050640613487194 PMC4040755 24917961

[9] Williams LJ Zolfaghari S Boushey RP. Complications of enterocutaneous fistulas and their management. Clin Colon Rectal Surg. 2010;23:209–20. 21886471 10.1055/s-0030-1263062 PMC2967321

[10] Amiot A Setakhr V Seksik P. Long-term outcome of enterocutaneous fistula in patients with Crohn’s disease treated with anti-TNF therapy: a cohort study from the GETAID. Am J Gastroenterol. 2014;109:1443–9. 25091063 10.1038/ajg.2014.183

[11] Moher D Liberati A Tetzlaff J. Preferred reporting items for systematic reviews and meta-analyses: the PRISMA statement. BMJ. 2009;339:b2535. 19622551 10.1136/bmj.b2535 PMC2714657

[12] Guyatt G Rennie D eds. Users’ Guides to the Medical Literature: A Manual for Evidence-Based Clinical Practice. Chicago, IL: American Medical Association, 2002:736.

[13] Sterne JA Hernan MA Reeves BC. ROBINS-I: a tool for assessing risk of bias in non-randomised studies of interventions. BMJ. 2016;355:i4919. 27733354 10.1136/bmj.i4919 PMC5062054

[14] Boyle M O’Connell PR Hyland J. Retrospective analysis of enterocutaneous fistula management in IBD. Ir J Med Sci. 2015;184:S235.

[15] Schwartz DA Tagarro I Carmen Diez M. Prevalence of fistulizing Crohn’s disease in the United States: estimate from a systematic literature review attempt and population-based database analysis. Inflamm Bowel Dis. 2019;25:1773–9. 31216573 10.1093/ibd/izz056 PMC6799946

[16] Bell SJ Williams AB Wiesel P. The clinical course of fistulating Crohn’s disease. Aliment Pharmacol Ther. 2003;17:1145–51. 12752351 10.1046/j.1365-2036.2003.01561.x

[17] Gyorki DE Brooks CE Gett R. Enterocutaneous fistula: a single-centre experience. ANZ J Surg. 2010;80:178–81. 20575922 10.1111/j.1445-2197.2009.05086.x

[18] Yoon YS Yu CS Yang SK. Intra-abdominal fistulas in surgically treated Crohn’s disease patients. World J Surg. 2010;34:1924–9. 20372893 10.1007/s00268-010-0568-3

[19] Li G Ren J Wang G. Preoperative exclusive enteral nutrition reduces the postoperative septic complications of fistulizing Crohn’s disease. Eur J Clin Nutr. 2014;68:441–6. 24549026 10.1038/ejcn.2014.16

[20] Tuma F Crespi Z Wolff CJ. Enterocutaneous fistula: a simplified clinical approach. Cureus. 2020;12:e7789. 32461860 10.7759/cureus.7789 PMC7243661

