# Supplementary material for: A systematic review of epidemiology and outcomes of Crohn’s disease-related enterocutaneous fistulas
Source: Medicine (Baltimore). 2022 Nov 11;101(45):e30963. doi: 10.1097/MD.0000000000030963 (PMC10662878; doi:10.1097/MD.0000000000030963)
Supplement: Supplementary file 1 [file medi-101-e30963-s001.pdf]

Supplemental Digital Content (Table S1). PubMed Search Strategy conducted on March 25, 2020

Row	Search concept	Terms
**Conditions**		
#1	**Condition 1**: Complex cryptoglandular fistulas (CCF) and/or cryptoglandular fistulas (CF)	((cryptoglandular[Title/Abstract] OR cryptogland*[Title/Abstract] OR horseshoe[Title/Abstract] OR branching[Title/Abstract]) AND<br><br>("Fistula"[MeSH] OR "fistula"[Title/Abstract] OR fistul*[Title/Abstract])) OR<br><br>((mid[Title/Abstract] OR high[Title/Abstract]) AND (transphincteric[Title/Abstract] OR trans-sphincteric[Title/Abstract] OR transsphincteric[Title/Abstract]) AND ("Fistula"[MeSH] OR "fistula"[Title/Abstract] OR fistul*[Title/Abstract]))
#2	**Condition 2a**: Rectovaginal fistula	"Rectovaginal Fistula"[MeSH] OR<br><br>(("rectovaginal"[Title/Abstract] OR "recto-vaginal"[Title/Abstract]) AND<br><br>("Fistula"[MeSH] OR "fistula"[Title/Abstract] OR fistul*[Title/Abstract]))
	**Condition 2b**: Anovaginal fistula	(("anovaginal"[Title/Abstract] OR "ano-vaginal"[Title/Abstract]) AND<br><br>("Fistula"[MeSH] OR "fistula"[Title/Abstract] OR fistul*[Title/Abstract]))

	**Condition 2c**: Enterocutaneous fistula	((“enterocutaneous”[Title/Abstract] OR “entero-cutaneous”[Title/Abstract]) AND<br><br>(“Fistula”[MeSH] OR “fistula”[Title/Abstract] OR fistul*[Title/Abstract]))
	**Condition 2 combined**<br><br>2a OR 2b OR 2c	(((“Rectovaginal Fistula”[MeSH] OR ((“rectovaginal”[Title/Abstract] OR “recto-vaginal”[Title/Abstract]) AND (“Fistula”[MeSH] OR “fistula”[Title/Abstract] OR fistul*[Title/Abstract])))) OR (((“anovaginal”[Title/Abstract] OR “ano-vaginal”[Title/Abstract]) AND (“Fistula”[MeSH] OR “fistula”[Title/Abstract] OR fistul*[Title/Abstract])))) OR (((“enterocutaneous”[Title/Abstract] OR “entero-cutaneous”[Title/Abstract]) AND (“Fistula”[MeSH] OR “fistula”[Title/Abstract] OR fistul*[Title/Abstract])))
#3	Crohn’s terms	“Crohn disease”[MeSH] OR crohn*[Title/Abstract]
#4	Fistula<br><br>(for title filter)	Fist*[Title]
**Real-World Data Terms**		
#5	Observational studies and real-world data	(“Cohort Studies”[MeSH] OR “Cross-Sectional Studies”[MeSH] OR “Longitudinal Studies”[MeSH] OR “Prospective Studies”[MeSH] OR “case-control study”[MeSH] OR “Registries”[MeSH] OR “Electronic Health Records”[MeSH] OR “Administrative Claims, Healthcare”[MeSH] OR “Observational Study” [Publication Type] OR

		cohort[Title/Abstract] OR cohort*[Title/Abstract] OR cross-sectional[Title/Abstract] OR cross-section*[Title/Abstract] OR longitudinal[Title/Abstract] OR longitud*[Title/Abstract] OR prospective[Title/Abstract] OR prospect*[Title/Abstract] OR retrospective[Title/Abstract] OR observational[Title/Abstract] OR observation*[Title/Abstract] OR registry[Title/Abstract] OR “electronic medical record”[Title/Abstract] OR “medical record”[Title/Abstract] OR “electronic health record”[Title/Abstract] OR “health record”[Title/Abstract] OR (claims[Title/Abstract] OR “claims data”[Title/Abstract] OR “administrative claims”[Title/Abstract] OR “systematic review”[Title/Abstract] OR “systematic literature review”[Title/Abstract] OR “meta-analysis”[Title/Abstract])
**Queries of Interest**		
#6	Measures of occurrence/association	“Epidemiology”[MeSH] OR “Pharmacoepidemiology”[MeSH] OR<br><br>epidemiolog*[Title/Abstract] OR pharmacoepidemiolog*[Title/Abstract] OR<br><br>prevalence[Title/Abstract] OR incidence[Title/Abstract] OR risk[Title/Abstract] OR rate[Title/Abstract] OR rates[Title/Abstract] OR proportion[Title/Abstract] OR proportions[Title/Abstract] OR frequency[Title/Abstract] OR frequencies[Title/Abstract]
#7	Healthcare resource utilization and costs	“Costs and Cost Analysis”[MeSH] OR “Economics”[MeSH] OR “Health Expenditures”[MeSH] OR “Cost of Illness”[MeSH] OR “Cost-Benefit Analysis”[MeSH] OR

		"Health Care Costs"[MeSH] OR "Direct Service Costs"[MeSH] OR "Hospital Costs"[MeSH] OR "Drug Costs"[MeSH] OR "Health Resources"[MeSH] OR "Resource Allocation"[MeSH] OR<br><br>"cost"[Title/Abstract] OR "costs"[Title/Abstract] OR "cost analysis"[Title/Abstract] OR "resource use"[Title/Abstract] OR "resource utilization"[Title/Abstract] OR expenditure[Title/Abstract] OR expenditures[Title/Abstract] OR economic[Title/Abstract] OR economics[Title/Abstract]
#8	Treatment patterns	"Immunosuppression"[MeSH] OR<br><br>"Immunosuppressive Agents"[MeSH] OR "Immunosuppressive Agents" [Pharmacological Action] OR "Anti-Bacterial Agents"[MeSH] OR "Anti-Bacterial Agents" [Pharmacological Action] OR "Antibodies, Monoclonal"[MeSH] OR "Surgical Procedures, Operative"[MeSH] OR "surgery" [Subheading] OR "Ostomy"[MeSH] OR<br><br>(immunosuppress*[Title/Abstract] OR antibiotic[Title/Abstract] OR antibiotics[Title/Abstract] OR corticosteroid*[Title/Abstract] OR prednisone[Title/Abstract] OR methotrexate[Title/Abstract] OR anti-TNF[Title/Abstract] OR "anti-tumor necrosis factor"[Title/Abstract] OR infliximab[Title/Abstract] OR adalimumab[Title/Abstract] OR "certolizumab pegol"[Title/Abstract] OR certolizumab[Title/Abstract] OR "monoclonal antibody"[Title/Abstract] OR "monoclonal antibodies"[Title/Abstract] OR natalizumab[Title/Abstract] OR vedolizumab[Title/Abstract] OR "interleukin

		antagonist"[Title/Abstract] OR "interleukin antagonists"[Title/Abstract] OR "interleukin inhibitor"[Title/Abstract] OR "interleukin inhibitors"[Title/Abstract] OR ustekinumab[Title/Abstract] OR<br><br>cyclosporine[Title/Abstract] OR tacrolimus[Title/Abstract] OR<br><br>surgery[Title/Abstract] OR Fistulotomy[Title/Abstract] OR "ligation of the intersphincteric fistula tract"[Title/Abstract] OR LIFT[Title/Abstract] OR ligation*[Title/Abstract] OR seton[Title/Abstract] OR "cutting seton"[Title/Abstract] OR "fibrosing seton"[Title/Abstract] OR "drainage seton" OR "seton placement"[Title/Abstract] OR flap[Title/Abstract] OR "advancement flap"[Title/Abstract] OR "advancement flaps"[Title/Abstract] OR ablation[Title/Abstract] OR "fibrin glue"[Title/Abstract] OR "bioprosthetic plug"[Title/Abstract] OR "anal fistula plug"[Title/Abstract] OR plug[Title/Abstract] OR plugs[Title/Abstract] OR sealant [Title/Abstract] OR<br><br>sealants[Title/Abstract] OR "surgical reconstruction"[Title/Abstract] OR "sphincteric reconstruction"[Title/Abstract] OR sphincterotomy[Title/Abstract] OR sphincteroplasty[Title/Abstract] OR proctectomy[Title/Abstract] OR diversion[Title/Abstract] OR therapy[Title/Abstract] OR therapeutic*[Title/Abstract] OR pattern*[Title/Abstract] OR "treatment pattern" [Title/Abstract] OR "treatment patterns" [Title/Abstract] OR "ostomy"[Title/Abstract] OR ostomies[Title/Abstract]OR enterostomy[Title/Abstract] OR enterostomies[Title/Abstract]

		OR cecostomy[Title/Abstract] OR cecostomies[Title/Abstract] OR colostomy[Title/Abstract] OR colostomies[Title/Abstract] OR duodenostomy[Title/Abstract] OR<br><br>duodenostomies[Title/Abstract] OR ileostomy[Title/Abstract] OR ileostomies[Title/Abstract] OR jejunostomy[Title/Abstract] OR jejunostomies[Title/Abstract])
#9	Clinical outcomes	“Treatment Outcome”[MeSH] OR<br><br>((“healing”[Title/Abstract] OR “success”[Title/Abstract] OR “response”[Title/Abstract] OR “recurrence”[Title/Abstract] OR “remission”[Title/Abstract] OR “incontinence”[Title/Abstract]) AND (“rate”[Title/Abstract] OR “frequency”[Title/Abstract] OR “proportion”[Title/Abstract])) OR<br><br>“closure”[Title/Abstract] OR “recurrence”[Title/Abstract] OR “recurrence risk”[Title/Abstract] OR “remission”[Title/Abstract] OR “remission risk”[Title/Abstract] OR “incontinence”[Title/Abstract] OR “incontinence risk”[Title/Abstract] OR “clinical response”[Title/Abstract] OR “clinical remission”[Title/Abstract]
#10	Patient-reported outcomes	“Patient Reported Outcome Measures”[MeSH] OR “Fecal Incontinence”[MeSH] OR<br><br>(“patient reported outcomes”[Title/Abstract] OR “clinical outcome”[Title/Abstract] OR “Crohn’s Disease Activity Index”[Title/Abstract] OR CDAI[Title/Abstract] OR “IBD questionnaire”[Title/Abstract] OR “IBDQ”[Title/Abstract] OR

		“inflammatory bowel disease questionnaire”[Title/Abstract] OR “Patient reported outcome measures”[Title/Abstract] OR PROMs[Title/Abstract] OR “perianal disease activity index”[Title/Abstract] OR “PDAI”[Title/Abstract] OR “CDAI”[Title/Abstract] OR “anal pain”[Title/Abstract] OR pain[Title/Abstract] OR “fecal incontinence”[Title/Abstract] OR “faecal incontinence”[Title/Abstract] OR “bowel incontinence”[Title/Abstract] OR “fecal soiling”[Title/Abstract] OR “faecal soiling”[Title/Abstract] OR “leakage”[Title/Abstract] OR “discharge”[Title/Abstract] OR “soiling”[Title/Abstract] OR “soil”[Title/Abstract] OR “soiled”[Title/Abstract] OR “pads”[Title/Abstract] OR “EQ-5D”[Title/Abstract] OR “EuroQol”[Title/Abstract] OR “EuroQol-5D”[Title/Abstract] OR “intercourse”[Title/Abstract] OR “sexual activity”[Title/Abstract] OR “Wexner Scale of incontinence”[Title/Abstract] OR “Revised Fecal Incontinence Scale”[Title/Abstract] OR “Revised Faecal Incontinence Scale”[Title/Abstract] OR “RFIS”[Title/Abstract])
**Combination Searches**		
#11	Condition 2 + Crohn’s	(#2a OR 2b OR 2c) AND #3
#12	Combined Queries	#6 OR #7 OR #8 OR #9 OR #10
**SEARCH RESULTS**		
#13	Condition 2 + Combined Queries + RWD + filters	#11 AND #12 AND #5 +English, human, 10 years filters

#14	Condition 1 + Combined Queries + RWD + filters	#1 AND #12 AND #5 +English, human, 10 years filters
#15	Final search including fistula title search	(#13 OR #14) AND #4
