# Supplementary material for: A systematic review of epidemiology and outcomes of Crohn’s disease-related enterocutaneous fistulas
Source: Medicine (Baltimore). 2022 Nov 11;101(45):e30963. doi: 10.1097/MD.0000000000030963 (PMC10662878; doi:10.1097/MD.0000000000030963)
Supplement: Supplementary file 2 [file medi-101-e30963-s002.pdf]

Supplemental Digital Content (Table S2). Embase Search Strategy conducted on March 25, 2020

Row	Search concept	Terms
**Conditions**		
#1	**Condition 1**: Complex cryptoglandular fistulas (CCF) and/or cryptoglandular fistulas (CF)	'cryptoglandular fistula':ab,ti,kw OR<br><br>((cryptoglandular:ti,ab,kw OR horseshoe:ab,ti,kw OR branching:ab,ti,kw) AND fistul*:ti,ab,kw) OR<br><br>((mid:ab,ti,kw OR high:ab,ti,kw) AND (transphincteric:ab,ti,kw OR trans-sphincteric:ab,ti,kw OR transsphincteric:ab,ti,kw) AND fistul*:ti,ab,kw)
#2	**Condition 2**:<br><br>Non-perianal Crohn's fistula (NPCF):<br><br>rectovaginal fistulas (RVF), ano-vaginal fistulas (AVF),<br><br>enterocutaneous fistulas (ECF)	'rectovaginal fistula':ti,ab,kw OR 'anovaginal fistula':ti,ab,kw OR 'enterocutaneous fistula':ti,ab,kw OR<br><br>((rectovaginal:ab,ti,kw OR recto-vaginal:ab,ti,kw OR anovaginal:ab,ti,kw OR ano-vaginal:ab,ti,kw OR enterocutaneous:ab,ti,kw OR entero-cutaneous:ab,ti,kw) AND fistul*:ab,ti,kw)
#3	Crohn's terms	'crohn disease':ab,ti,kw OR crohn*:ab,ti,kw
#4	Fistula<br><br>(for title filter)	Fist*:ti
**Real-World Data Terms**		

#5	Observational studies and real-world data	‘cohort analysis’:ab,ti,kw OR ‘cross-sectional study’:ab,ti,kw OR ‘cross sectional analysis’:ab,ti,kw OR ‘longitudinal study’:ab,ti,kw OR ‘prospective study’:ab,ti,kw OR ‘case control study’:ab,ti,kw OR ‘electronic medical record’:ab,ti,kw OR ‘electronic health record’:ab,ti,kw OR ‘administrative claims’:ab,ti,kw OR ‘observational study’:ab,ti,kw OR<br><br>‘cohort’:ab,ti,kw OR cohort*:ab,ti,kw OR ‘cross-sectional’:ab,ti,kw OR ‘longitudinal’:ab,ti,kw OR ‘prospective’:ab,ti,kw OR ‘retrospective’:ab,ti,kw OR ‘observational’:ab,ti,kw OR ‘registry’:ab,ti,kw OR ‘register’:ab,ti,kw OR ‘registries’:ab,ti,kw OR ‘medical record’:ab,ti,kw OR ‘health record’:ab,ti,kw OR ‘medical record’:ab,ti,kw OR ‘claims data’:ab,ti,kw OR ‘administrative claims’:ab,ti,kw OR ‘claims’:ab,ti,kw OR ‘systematic review’:ab,ti,kw OR ‘systematic literature review’:ab,ti,kw OR ‘meta-analysis’:ab,ti,kw OR ‘meta analysis’:ab,ti,kw
**Queries of Interest**		
#6	Measures of occurrence/association	epidemiolog*:ti,ab,kw OR pharmacoepidemiolog*:ti,ab,kw OR prevalence:ti,ab,kw OR incidence:ti,ab,kw OR rate:ti,ab,kw OR rates:ti,ab,kw OR risk:ti,ab,kw OR proportion:ti,ab,kw OR proportions:ti,ab,kw OR frequency:ti,ab,kw OR frequencies:ti,ab,kw
#7	Healthcare resource utilization and costs	‘resource utilization’:ti,ab,kw OR ‘resource use’:ti,ab,kw OR ‘resource allocation’:ti,ab,kw OR ‘health resources’:ti,ab,kw OR ‘health care utilization’:ti,ab,kw OR cost:ti,ab,kw OR ‘cost analysis’:ti,ab,kw OR economic:ti,ab,kw OR expenditures:ti,ab,kw OR ‘health care cost’:ti,ab,kw

#8	Treatment patterns	‘immunosuppressive agent’:ti,ab,kw OR ‘antibiotic drug’:ti,ab,kw OR<br><br>corticosteroid:ti,ab,kw OR prednisone:ti,ab,kw OR methotrexate:ti,ab,kw OR ‘anti tnf’:ti,ab,kw OR ‘tumor necrosis factor inhibitor’:ti,ab,kw OR infliximab:ti,ab,kw OR adalimumab:ti,ab,kw OR ‘certolizumab pegol’:ti,ab,kw OR ‘certolizumab’:ti,ab,kw OR ‘monoclonal antibody’:ti,ab,kw OR natalizumab:ti,ab,kw OR vedolizumab:ti,ab,kw OR ‘interleukin antagonist’ OR ‘interleukin inhibitor’:ti,ab,kw OR ustekinumab:ti,ab,kw OR<br><br>surgery:ti,ab,kw OR surgical:ti,ab,kw OR fistulotomy:ti,ab,kw OR sphincterotomy:ti,ab,kw OR sphincteroplasty:ti,ab,kw OR proctectomy:ti,ab,kw OR ligation:ti,ab,kw OR ‘ligation of the intersphincteric fistula tract’:ti,ab,kw OR lift:ti,ab,kw OR seton:ti,ab,kw OR ‘seton placement’:ti,ab,kw OR flap:ti,ab,kw OR ‘advancement flap’:ti,ab,kw OR ablation:ti,ab,kw OR ‘fibrin glue’:ti,ab,kw OR ‘bioprosthetic plug’:ti,ab,kw OR patterns:ti,ab,kw OR ‘treatment pattern’:ti,ab,kw OR ‘treatment patterns’:ti,ab,kw OR therapy:ti,ab,kw OR therapeutic:ti,ab,kw OR ‘cutting seton’:ti,ab,kw OR ‘fibrosing seton’:ti,ab,kw OR ‘drainage seton’:ti,ab,kw OR ‘sphincteric reconstruction’:ti,ab,kw OR ‘anal fistula plug’:ti,ab,kw OR sealant:ti,ab,kw OR ‘surgical reconstruction’:ti,ab,kw OR<br><br>ostomy:ti,ab,kw OR ostomies:ti,ab,kw OR enterostomy:ti,ab,kw OR enterostomies:ti,ab,kw OR cecostomy:ti,ab,kw OR cecostomies:ti,ab,kw OR colostomy:ti,ab,kw OR colostomies:ti,ab,kw OR duodenostomy:ti,ab,kw OR duodenostomies:ti,ab,kw OR

		ileostomy:ti,ab,kw OR ileostomies:ti,ab,kw OR jejunostomy:ti,ab,kw OR jejunostomies:ti,ab,kw
#9	Clinical outcomes	'clinical outcome':ti,ab,kw OR 'clinical response':ti,ab,kw OR remission:ti,ab,kw OR recurrence:ti,ab,kw OR closure:ti,ab,kw OR 'incontinence':ti,ab,kw OR<br><br>'healing rate':ti,ab,kw OR 'success rate':ti,ab,kw OR<br><br>'response rate':ti,ab,kw OR 'recurrence rate':ti,ab,kw OR 'recurrence risk':ti,ab,kw OR 'remission rate':ti,ab,kw OR 'remission risk':ti,ab,kw OR 'incontinence rate':ti,ab,kw OR 'incontinence risk':ti,ab,kw
#10	Patient-reported outcomes	'patient-reported outcome':ti,ab,kw OR 'crohns disease patient-reported outcomes signs':ti,ab,kw OR 'cd pro':ti,ab,kw OR 'crohn disease activity index':ti,ab,kw OR cdai:ti,ab,kw OR 'ibd questionnaire':ti,ab,kw OR ibdq:ti,ab,kw OR 'inflammatory bowel disease questionnaire':ti,ab,kw OR 'patient reported outcome measure':ti,ab,kw OR proms:ti,ab,kw OR 'perianal disease activity index':ti,ab,kw OR PDAI:ti,ab,kw OR discharge:ti,ab,kw OR soiling:ti,ab,kw OR soil:ti,ab,kw OR soiled:ti,ab,kw OR pads:ti,ab,kw OR 'fecal incontinence':ti,ab,kw OR 'faecal incontinence':ti,ab,kw OR 'bowel incontinence':ti,ab,kw OR 'fecal soiling':ti,ab,kw OR 'faecal soiling':ti,ab,kw OR 'EQ-5D':ti,ab,kw OR 'EuroQol':ti,ab,kw OR 'EuroQol-5D':ti,ab,kw OR intercourse:ti,ab,kw OR 'sexual behavior':ti,ab,kw OR 'sexual activity':ti,ab,kw OR 'Wexner Scale of incontinence':ti,ab,kw OR 'Revised fecal incontinence scale':ti,ab,kw OR 'Revised faecal incontinence

		scale':ti,ab,kw OR 'RFIS':ti,ab,kw OR 'anal pain':ti,ab,kw OR pain:ti,ab,kw OR 'leakage':ti,ab,kw
**Combination Searches**		
#11	Condition 2 + Crohn's	#2 AND #3
#12	Combined Queries	#6 OR #7 OR #8 OR #9 OR #10
**SEARCH RESULTS**		
#13	Condition 2 + Combined Queries + RWD + filters	#11 AND #12 AND #5 +English, human, 10 years filters
#14	Condition 1 + Combined Queries + RWD + filters	#1 AND #12 AND #5 +English, human, 10 years filters
#15	Final search including fistula title search	(#13 OR #14) AND #4
