# Supplementary material for: A systematic review of epidemiology and outcomes of Crohn’s disease-related enterocutaneous fistulas
Source: Medicine (Baltimore). 2022 Nov 11;101(45):e30963. doi: 10.1097/MD.0000000000030963 (PMC10662878; doi:10.1097/MD.0000000000030963)
Supplement: Supplementary file 3 [file medi-101-e30963-s003.pdf]

Supplemental Digital Content (Table S3). Population, intervention, comparison, outcomes, time, and study design (PICOTS) criteria

**Inclusion criteria**	
Population(s)	• Patients with Crohn's Disease-related ECF<br>• No restrictions regarding other demographic characteristics
Intervention(s)	• No restrictions for assessment of incidence/prevalence or HCRU/costs<br>• Treatment patterns, ClinROs and PROs will focus on the following interventions:<br>- Pharmacologic: antibiotics, immunosuppressants, corticosteroids, prednisone, methotrexate, anti-tumor necrosis factor (infliximab, adalimumab, certolizumab pegol), other monoclonal antibody (natalizumab, vedolizumab), interleukin antagonist (ustekinumab)<br>- Surgical: fistulotomy, ligation of the intersphincteric fistula tract, cutting seton, fibrosing seton, drainage seton, sphincteric reconstruction, flap, ablation, fibrin glue, anal fistula plug, bioprosthetic plug, surgical reconstruction, sphincterotomy, sphincteroplasty, proctectomy, diversion, ostomy
Comparison(s)	• No restrictions on comparators for assessment of incidence/prevalence or HCRU/costs<br>• Assessment of treatment patterns in studies that include the pharmacologic or surgical interventions listed above<br>• Clinical and patient-reported outcomes in single arm or comparator studies assessing the interventions listed above
Outcome(s)	• Incidence and prevalence<br>• HCRU: Outpatient physician visits, inpatient/hospital admissions, emergency room visits, laboratory tests, diagnostic and therapeutic tests, length of stay<br>• Direct and indirect costs<br>• Treatment patterns: proportion of patients utilizing prespecified surgery or pharmacologic treatment

	• Clinical outcomes: healing rate, success rate, response rate, recurrence rate, fistula closure, clinical response, clinical remission<br>• Specific PRO instruments of interest: Crohn's Disease Activity Index (CDAI), Inflammatory Bowel Disease Questionnaire (IBDQ), 5-dimension EuroQol questionnaire (EQ-5D)<br>• Other PRO categories: pain
Time	• No restriction on duration of illness or treatment<br>• Publications within the past 10 years
Study design	• Observational studies: case-control studies, retrospective or prospective cohort studies/registries, cross-sectional studies<br>• Pooled data excluded; however, studies identified in those reports would be reviewed as part of the manual search for inclusion. Other publications that are not original research, clinical trials, or case reports/series[†] are excluded
Other – language	• English language

[†]Case series are included if they meet prespecified criteria for cohort design.

CDAI, Crohn's Disease Activity Index; ClinRO, clinical-reported outcome; ECF, enterocutaneous fistulas; EQ-5D, 5-dimension EuroQol questionnaire; HCRU, healthcare resource utilization; PRO, patient-reported outcome.
